# Clinical experience on patient‐specific quality assurance for CBCT‐based online adaptive treatment plan

**DOI:** 10.1002/acm2.13918

**Published:** 2023-02-02

**Authors:** Chenyang Shen, Liyuan Chen, Xinran Zhong, Yesenia Gonzalez, Justin Visak, Boyu Meng, Enobong Inam, David Parsons, Andrew Godley, Steve Jiang, Bin Cai, Mu‐Han Lin

**Affiliations:** ^1^ Department of Radiation Oncology University of Texas Southwestern Medical Center Dallas Texas USA

**Keywords:** adaptive radiotherapy, patient‐specific QA

## Abstract

**Purpose:**

Ethos CBCT‐based adaptive radiotherapy (ART) system can generate an online adaptive plan by re‐optimizing the initial reference plan based on the patient anatomy at the treatment. The optimization process is fully automated without any room for human intervention. Due to the change in anatomy, the ART plan can be significantly different from the initial plan in terms of plan parameters such as the aperture shapes and number of monitor units (MUs). In this study, we investigated the feasibility of using calculation‐based patient specific QA for ART plans in conjunction with measurement‐based and calculation‐based QA for initial plans to establish an action level for the online ART patient‐specific QA.

**Methods:**

A cohort of 98 cases treated on CBCT‐based ART system were collected for this study. We performed measurement‐based QA using ArcCheck and calculation‐based QA using Mobius for both the initial plan and the ART plan for analysis. For online the ART plan, Mobius calculation was conducted prior to the delivery, while ArcCheck measurement was delivered on the same day after the treatment. We first investigated the modulation factors (MFs) and MU numbers of the initial plans and ART plans, respectively. The γ passing rates of initial and ART plan QA were analyzed. Then action limits were derived for QA calculation and measurement for both initial and online ART plans, respectively, from 30 randomly selected patient cases, and were evaluated using the other 68 patient cases.

**Results:**

The difference in MF between initial plan and ART‐plan was 12.9% ± 12.7% which demonstrates their significant difference in plan parameters. Based on the patient QA results, pre‐treatment calculation and measurement results are generally well aligned with ArcCheck measurement results for online ART plans, illustrating their feasibility as an indicator of failure in online ART QA measurements. Furthermore, using 30 randomly selected patient cases, the γ analysis action limit derived for initial plans and ART plans are 89.6% and 90.4% in ArcCheck QA (2%/2 mm) and are 92.4% and 93.6% in Mobius QA(3%/2 mm), respectively. According to the calculated action limits, the ArcCheck measurements for all the initial and ART plans passed QA successfully while the Mobius calculation action limits flagged seven and four failure cases respectively for initial plans and ART plans, respectively.

**Conclusion:**

An ART plan can be substantially different from the initial plan, and therefore a separate session of ART plan QA is needed to ensure treatment safety and quality. The pre‐treatment QA calculation via Mobius can serve as a reliable indicator of failure in online ART plan QA. However, given that Ethos ART system is still relatively new, ArcCheck measurement of initial plan is still in practice. It may be skipped as we gain more experience and have better understanding of the system.

## 1 INTRODUCTION

**FIGURE 1:**
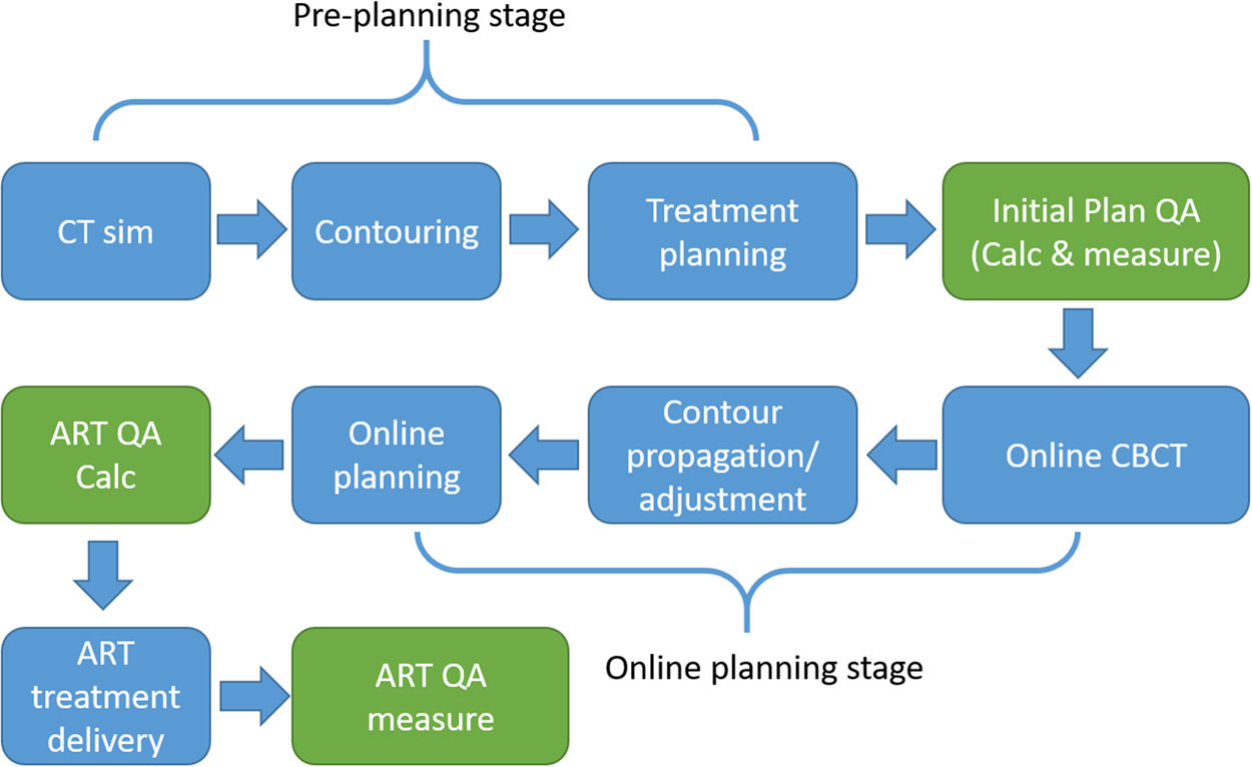
A complete adaptive radiation therapy workflow. The quality assurance steps are highlighted in green

Empowered by the recent advancements in artificial intelligence,^1^, ^2^ online adaptive radiotherapy (ART) has become an emerging treatment technique to improve quality and accuracy of radiotherapy. Ethos ART system (Varian Medical System, Inc., Palo Alto, CA) is a commercially available platform dedicated to online adapting an initial treatment plan to the patient anatomy.^3^ A complete ART workflow on Ethos can be found in Figure 1. The planning process of Ethos ART consists of two stages, for example, a pre‐planning stage and an online ART planning stage. The pre‐planning stage is remarkably similar to the conventional radiotherapy treatment planning process.^4^ Based on the CT simulation and the corresponding contours of the treatment targets and organs at risk (OARs), a planner will set up planning objectives and constraints, and carefully adjust them to generate a high‐quality initial plan as a starting point for online ART planning. The final planning objectives/parameters from the initial reference plan generation are used as input to optimize the online ART plan. During online ART, a cone‐beam CT (CBCT) is acquired prior to treatment for automatic generation of the synthetic CT and contours. Clinicians will review the contours and edit them as needed. The ART plan is optimized with the planning objectives and constraints defined for initial plan generation. This online optimization process is fully automated with no human intervention allowed. Hence, planners do not have control of detailed plan parameters, for example, plan normalization value, aperture number and modulation, in the online ART session.

Due to the potential anatomical change from the CT simulation to treatment,^5^, ^6^ an optimized ART plan can be significantly distinct from the initial plan in terms of the aperture shapes and number of monitor units (MUs). A separate patient‐specific intensity‐modulated radiotherapy quality assurance (QA)^7^, ^8^ needs to be performed specifically for the ART plan to ensure patient safety and treatment quality in the ART session. Conventional measurement‐based QA is performed to ensure the safety of the patient and success of the treatment as the measurement is typically performed at least one day prior to treatment.^8^ This approach is not feasible for the online ART plan as the patient remains on the treatment couch throughout the whole ART session. This potentially mitigates change in patient anatomy and positioning. Therefore, it is critically important to seek reliable alternative patient‐specific QA approaches. In this study, we investigated the possibility of removing measurement‐based QA for ART plan by combining measurement‐based and calculation‐based QA for the initial plan, with the calculation‐based patient‐specific QA for ART plan to determine pass/failure criteria. We analyzed all the results from pre‐treatment QA calculations and measurements available and compared those with the post‐treatment ART QA measurements to facilitate a reliable prediction for ART plan QA pass/failure status prior to treating the patient.

## 2 METHOD

In this study, we have collected a cohort of 98 ART patient cases treated with Ethos ART in our institution. The detailed number of cases in each treatment site is listed in Table 1. All the patients are planned using fixed beam intensity‐modulated radiation therapy (IMRT) per our institutional protocol with the total number of beams for each plan ranges from 6 to 18. Among all the ART cases, 54 (55%) of them were treated using stereotactic body radiation therapy (SBRT). For each case, pre‐treatment QA measurement using ArcCheck (Sun Nuclear Corporation, Melbourne, FL) and QA calculation using Mobius3D (Varian Medical System, Inc., Palo Alto, CA) were both performed for the initial plan. For the online ART plan, we focused on the plan generated for the first online‐ART session for a fair comparison as different patients may have different numbers of ART sessions. Only Mobius3D calculation was conducted prior to treatment delivery. ArcCheck measurement was performed on the same day after the online ART treatment delivery.

**TABLE 1:** Numbers of cases in different treatment sites used in this study

Anatomical Sites	Central nerve system/Head and Neck	Thorax	Abdomen	Pelvis	Total
No. of Cases	14	39	18	27	98

Given the same optimization objectives were employed in both the initial and ART plans, we first evaluated the necessity of independent patient‐specific QA for the ART plans. We highlighted the differences between the initial plans and ARTs by comparing their modulation factors (MFs). As an ART plan and its corresponding initial plan are prescribed in exactly the same way, the difference in MF can serve as an indicator of different MLC apertures. Therefore, a significant difference in MF implies a non‐negligible difference between the initial and ART plan, revealing the need of an independent plan QA process for the online ART plan.

Then we investigated the patient‐specific QA results for the initial and ART plans. We employed the γ passing rates^9^, ^10^ as the evaluation metric to analyze the results in which the criteria for γ analysis was set as 2%/2 mm for ArcCheck measurements, and 3%/2 mm for Mobius calculation, both with 10% of prescription dose as the threshold to cut off the impact of low‐dose regions. Note that we picked stricter criteria of 2%/2 mm for ArcCheck γ analysis as compared to the commonly used 3%/2 mm since the γ results were mostly acceptable, that is, ≥90%. 2%/2 mm criteria were also employed in other dosimetric study in literature.^11^ The γ passing rates of the post‐treatment ArcCheck measurement for the ART plan and all the pre‐treatment measurements and calculations were analyzed. Furthermore, we established the action limit for all the measurement and calculation results of both initial and online ART plans based on TG‐218 recommendations.^8^ More precisely, for each patient‐specific QA test, we calculated a lower action limit based on:

(1) $$\mathrm{Lower}\;\mathrm{action}\;\mathrm{limit}\;=\bar{x}\;\;-\frac{\beta }{2}\sqrt{\sigma^{2}+{\left(\bar{x}-T\right)}^{2}}$$

*T* is the process target γ passing rate while σ^2^ and  $\bar{x}$ are the variance and mean, respectively. β is a constant and was set to be 6 in our study as recommended in TG‐218.^8^ The pass/fail status of different QA calculations and measurements for all patients in the cohort were then analyzed. Based on these results, we hope to establish the action limit for ART plan QA using all pretreatment results including that of both calculation and measurement QA for initial plan and ART plan calculation QA to eliminate the need of measurement‐based QA for ART plans.

## 3 RESULTS

**FIGURE 2:**
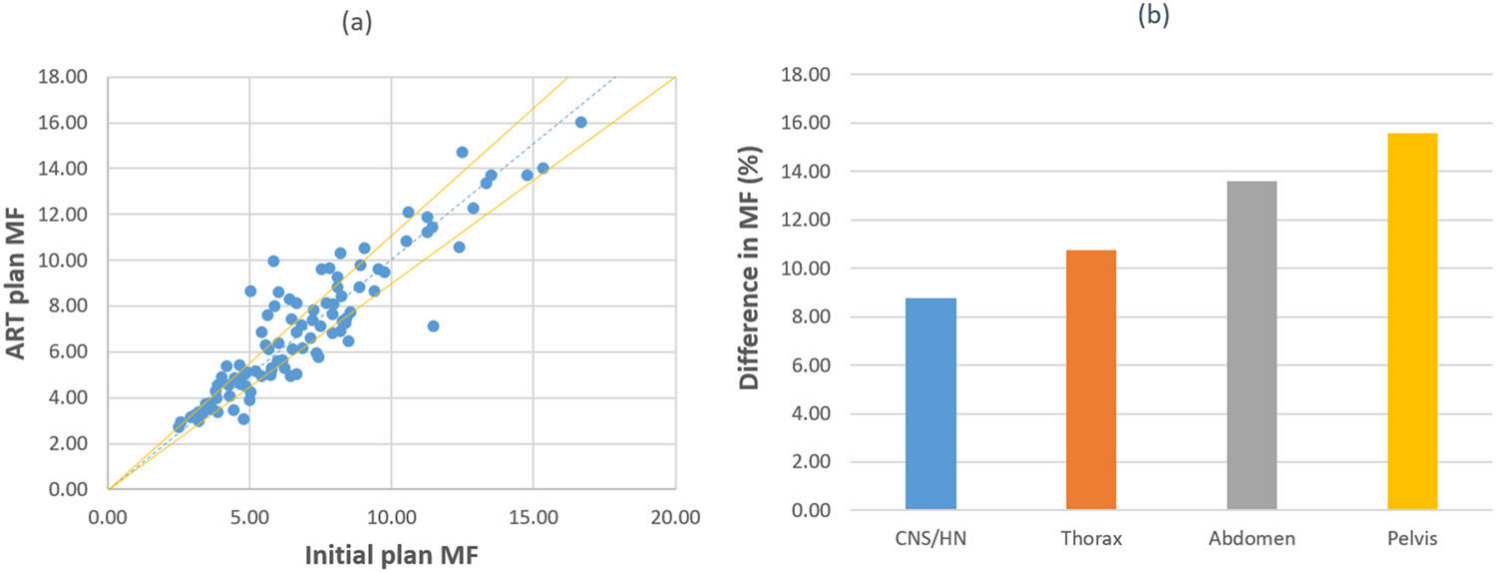
(a) MF comparison between the initial plans and online ART plans. The dashed blue line shows when ART plan MFs and initial plan MFs are equal, and yellow lines are ±10% deviated from the blue line. (b) MF difference between the initial plans and online ART plans in different treatment sites

The difference between an initial plan and its corresponding online ART plan can be simply evaluated by comparing their MFs since both plans are prescribed in an identical manner. Figure 2a compares the MF of initial and ART plan for each patient case in our study. Out of the total number of 98 cases in our patient cohort, difference in MF is ≥3% for 85 cases, and is ≥10% for 45 cases. More quantitative comparisons have been depicted in Figure 2b showing the average difference in MF for patient cases of different treatment sites. The smallest average difference of ∼8.8% was identified in central nerve system (CNS) and head and neck (HN) plans as compared to 15.6% of difference in pelvis plans. These results illustrate the fact that an ART plan can be distinct from the initial plan, and hence patient‐specific QA for ART plan is necessary to ensure patient safety and treatment quality.

**FIGURE 3:**
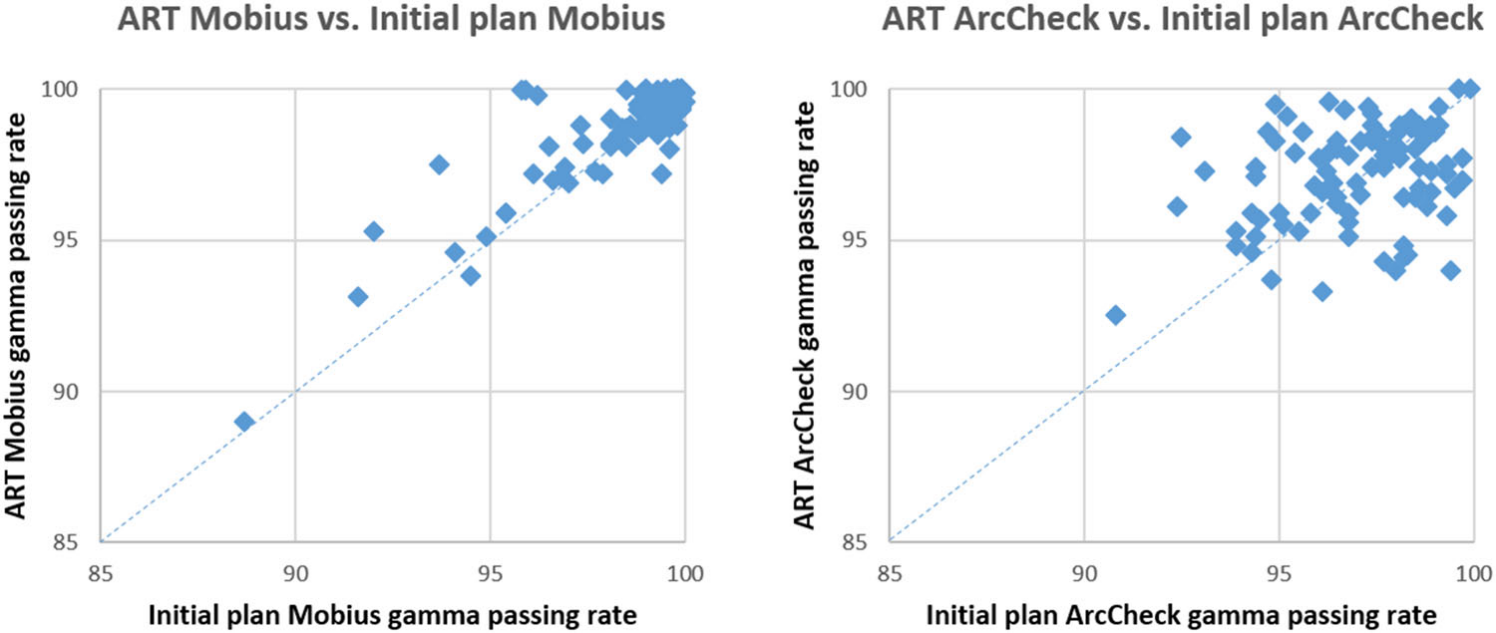
passing rate comparisons between the ART plans and the corresponding initial plans in Mobius (left) and ArcCheck (right) QA

In Figure 3 we compare the QA results of ART plans against their corresponding initial plans. The results from pre‐treatment QA tests agree well with the post‐treatment ArcCheck measurement results. The results showed that the online ART plans are quite robust with an exceptionally low chance of failure. More precisely, if we simply consider the commonly used action limit of 90% for γ passing rate, all the ART plans from our patient cohort passed ArcCheck QA measurements. Only one ART plan failed with <90% γ passing rate in Mobius calculation and the corresponding Mobius QA of the initial plan also failed.

**FIGURE 4:**
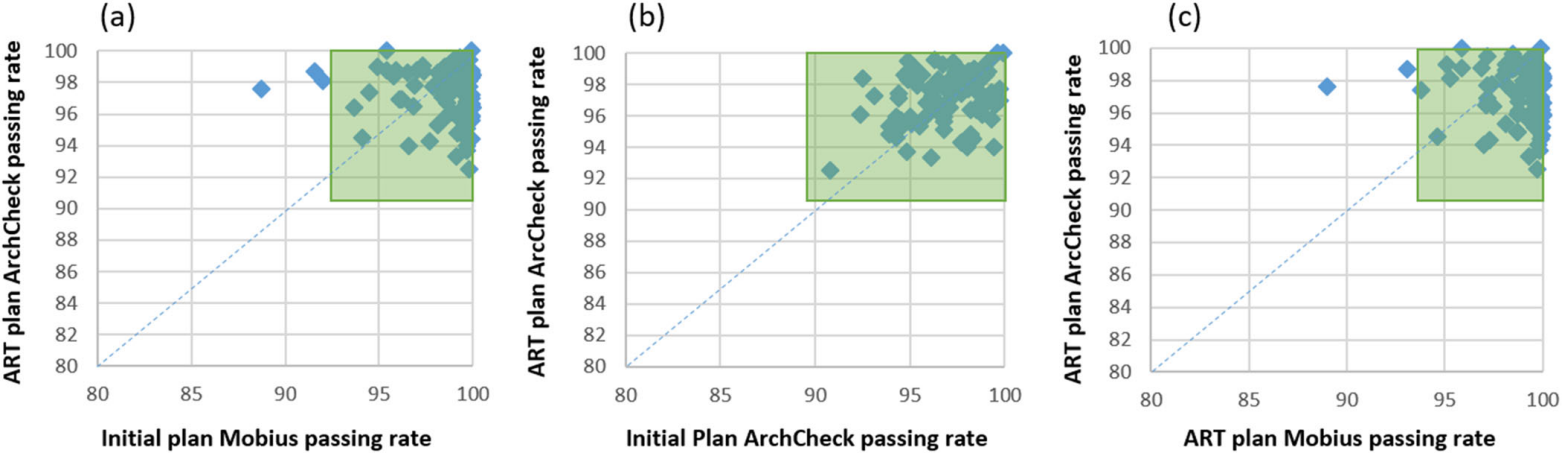
(a) Initial plan Mobius passing rate versus ART plan ArcCheck passing rate. (b) Initial plan ArcCheck passing rate versus ART plan ArcCheck passing rate. (c) ART plan Mobius passing rate vs. ART plan ArcCheck passing rate. The green boxes highlight the calculated action limits for different tests

Figure 4 plots the relation between the ART plan ArcCheck QA passing rate and all the QA measurement and calculation available prior to the treatment delivery. For each calculation‐ or measurement‐based QA, an action limit was derived following TG‐218. The resulting action levels for initial and ART plans are 89.6% and 90.4% in ArcCheck QA (2%/2 mm) and are 92.4% and 93.6% in Mobius QA (3%/2 mm), respectively, as highlighted in green in the zoom‐in plots in Figure 4. These action levels are reasonable compared to the action levels derived in other related dosimetric studies using ArcCheck^11^, ^12^ and Mobius.^13^, ^14^ According to the calculated action limits, the ArcCheck measurements for all the initial and ART plans passed QA successfully. While the Mobius calculation action limits flagged four and two failure cases respectively for initial and ART plans, respectively. The Mobius calculation seemed to be more sensitive to QA failure compared to ArcCheck measurements. Note that this may be partly due to the inhomogeneity correction in dose calculation as Mobius takes real human anatomy and heterogeneity into account in calculation while ArcCheck performs calculations and takes measurements in an almost uniform medium. In this regards, Mobius calculation can be considered as a more conservative QA compared to ArcCheck test for ART plan patient‐specific QA. In addition, as shown in the Figure 4b, the trend in ArcCheck measurements for initial plans generally agrees well with that of the ART plans, and hence the initial plan ArcCheck result has the potential to serve as a predictive indicator of ART plan ArcCheck measurement. These results illustrate that pre‐treatment QA tests, including Mobius results of both initial and ART plans, and ArcCheck measurement of initial plan, should be sufficient to ensure the treatment safety and quality of the online ART plan.

## 4 CONCLUSION AND DISCUSSIONS

In this study, we have analyzed the QA results from Mobius calculation and ArcCheck measurement performed for the initial and online ART plans generated for in the Ethos ART system in our institution. The results showed that an online ART plan can be substantially different from the initial plan, and therefore a separate ART plan QA is necessary to ensure treatment safety and quality.

Pre‐treatment measurement‐based QA is not feasible for online ART plan as the patient is always on the couch throughout the online ART session. Instead, we performed post‐treatment measurement and analyzed the results with those of initial plans and ART pre‐treatment calculation QA. Based on our analysis the ART planning process is overall quite robust with a low chance of failing QA. The QA results of initial plans and ART plans generally agree with each other.

Based on our observation, the Mobius calculation for the ART plan alone, which takes the inhomogeneity in patient body into account, is sufficient for patient‐specific QA of online ART plan generated by Ethos ART system. However, given that Ethos ART system is still relatively new, the understanding on the system characteristics and modeling is still extremely limited. In this regard, measurement‐based QA for the initial plan is still performed. Moreover, it is also worthwhile to mention that Mobius3D calculation software and Ethos ART systems are from the same vendor so the results might be biased. An independent secondary dose calculation to comprehensively verify the results is recommended as future studies before completely getting rid of the measurement‐based QA. As for now, pre‐treatment QA calculations via Mobius along with the QA calculation and measurement for the initial plan can serve as an indicator to flag failure in online ART plan QA.

## AUTHOR CONTRIBUTIONS

Chenyang Shen, Bin Cai, and Mu‐Han Lin designed the study. Chenyang Shen, Liyuan Chen, Xinran Zhong, Yesenia Gonzalez, Justin Visak, Boyu Meng, and Enobong Inam collected and analyzed data. David Parsons, Andrew Godley, and Steve Jiang provided clinical supervision and input to the study. Chenyang Shen and Mu‐Han Lin drafted the manuscript and all authors revised and approved the final manuscript.

## CONFLICT OF INTEREST

The authors have no relevant conflicts of interest to disclose.

## Data Availability

The data that support the findings of this study are available from the corresponding author upon reasonable request.
